# Evaluation of Solubility, and Volumetric and Morphological Alterations of Bioceramic Filling Material for Primary Teeth: A New Methodological Approach

**DOI:** 10.1155/2024/5945033

**Published:** 2024-06-24

**Authors:** Víctor M. Ochoa-Rodríguez, Hernán Coaguila-Llerena, Leandro Fernandes, Ana B. B. Solcia, Juliane M. Guerreiro-Tanomaru, Mário Tanomaru-Filho, Gisele Faria

**Affiliations:** ^1^ Department of Restorative Dentistry Araraquara School of Dentistry São Paulo State University—UNESP, Araraquara, São Paulo, Brazil; ^2^ Department of Endodontics School of Dentistry Peruvian University of Applied Sciences—UPC, Lima, Peru; ^3^ Department of Dental Materials and Prosthesis Araraquara School of Dentistry São Paulo State University—UNESP, Araraquara, São Paulo, Brazil

## Abstract

**Objective:**

To evaluate the solubility and the volumetric and morphological alterations of bioceramic filling material (Bio-CP) for primary teeth.

**Materials and Methods:**

Bio-CP, Calen thickened with zinc oxide (Calen-ZO), and with zinc oxide eugenol (ZOE) were placed in 1- or 2-mm-diameter polyethylene tubes and immersed in water or phosphate-buffered saline (PBS) for 30 days. The solubility (mass loss) was assessed using methodology modified from ISO 6876. Filling capacity, volumetric changes, and presence of voids were assessed by microcomputed tomography (micro-CT). The surface distribution of the chemical elements and the crystalline phases was evaluated by energy scattering X-ray scanning electron microscopy (SEM-EDX) and X-ray diffraction (XRD) to detect hydroxyapatite precipitate and components. The Shapiro–Wilk, Kruskal–Wallis, and Dunn's or two-way ANOVA and Tukey post hoc test were used (*α* = 0.05).

**Results:**

The solubility was ZOE > Calen-ZO = Bio-CP. Calen-ZO and Bio-CP were more soluble in water than in PBS. All the materials showed greater solubility in 2-mm tube diameter in both PBS and distilled water, except for Bio-CP in distilled water, which showed no difference between both tube diameters (1 and 2 mm). Only Calen-ZO and ZOE were analyzed by micro-CT, because Bio-CP separated into two phases during scanning. Calen-ZO had greater volumetric loss and presence of voids than ZOE in water, but there was no difference in PBS. The hydroxyapatite precipitate on the surface of Bio-CP and Calen-ZO was detected after immersion in PBS.

**Conclusion:**

Although Bio-CP had acceptable solubility and filling capacity, its composition did not allow a proper volumetric and void assessment. From a clinical perspective, Bio-CP has the potential to become a suitable material for root canal filling in primary teeth. Nonetheless, its composition must first be revised to achieve better chemical stability prior to its recommendation.

## 1. Introduction

Traditionally, calcium hydroxide- and zinc oxide eugenol (ZOE)-based materials are used for root canal filling of primary teeth. However, they do not meet all the requirements for an ideal filling material [1]. Comparatively, bioceramics (calcium silicate-based materials), used as sealers or repair materials on permanent teeth, have beneficial properties, such as alkaline pH, antibacterial activity, low contraction, stability in a biological environment, biocompatibility, and bioactivity [2].

Bio-C Pulpecto (Bio-CP; Angelus, Londrina, Paraná, Brazil) is the first bioceramic root filling material for primary teeth and is composed of ester glycol salicylate, titanium oxide, calcium tungstate, silicon dioxide, toluene sulfonamide, and calcium silicate (Angelus: product in development). From a biological point of view, Bio-CP showed cytocompatibility and the potential to induce mineralization comparable to Calen thickened with zinc oxide (Calen-ZO) in osteoblast-like cells (Saos-2) [3], as well as biocompatibility and mineralization induction in the subcutaneous tissue of rats, comparable to mineral trioxide aggregate (MTA) [4]. A recent study [3] revealed that Bio-CP has an alkaline pH, does not set, and has adequate radiopacity, that is, greater than 3 mm Al, as recommended by ISO 6876 [5]. Regarding Bio-CP solubility, there is only one study that evaluated this topic [6]; however, using a methodology, that may not reflect the clinical situation.

In the case of root filling materials for primary teeth, an important attribute is for solubility to be balanced, since the roots of primary teeth are physiologically resorbed. This means that the solubility of filling materials for primary teeth should not be high, to avoid the formation of gaps (voids), or low, to avoid their being retained in the tissue, hence causing inflammation, or impairing the eruption of the permanent successor [7].

The ISO 6876 [5] guidelines are recommended to evaluate the solubility of endodontic materials for permanent teeth. After a 50% longer than setting time period, the specimens should be weighed and immersed in distilled water for 24 hr, and then, after stabilization of the mass (dehydration), the specimens should be weighed again. However, this model guideline does not reproduce a clinical situation, in which the materials are placed in contact with the tissue fluids and/or blood during the root canal treatment and then exposed to solubilization for more than 24 hr [8]. For this reason, researchers have developed other methodologies using microcomputed tomography (micro-CT) to complement the evaluation of root canal sealer solubility [8]. According to this methodology, the root canals of permanent teeth are filled, and the teeth are immediately placed in the immersion medium [8]. The immersion time ranges from 7 to 30 days [9], and the immersion medium can be distilled water [9] or phosphate-buffered saline (PBS) [8]. In this model, the teeth are scanned using micro-CT immediately after filling and immersion, to allow the correlation of volumetric and morphological changes with solubility [8]. It has been reported that the solubility of calcium silicate-based sealers in contact with phosphate-containing solutions is significantly lower than their solubility in distilled water [10]. This could be attributed to precipitation of the mineral structure (identified as apatite-like) on the surface of these materials [11].

ISO 6876 methodology cannot be used to assess the solubility of filling materials for primary teeth [5] since many of these materials do not set. The present study included a novel approach to assess the solubility of these materials in different immersion media (PBS or distilled water) for a longer time than 24 hr to simulate a clinical situation. Currently, there is no defined protocol for performing solubility analysis (expressed as mass loss) of primary tooth filling materials or for evaluating volumetric or morphological changes by using micro-CT. Notably, the absence of such protocols can compromise the scientific impact of the studies; therefore, a methodology must be developed to address these issues.

Thus, the aim of the study was to evaluate (1) the solubility (% mass loss) of filling materials for primary teeth, using a modified methodology derived from ISO 6876, (2) the volumetric alteration of the filling capacity, and (3) the presence of voids in the three filling materials, using micro-CT. In parallel, other factors studied included the effect size of the polyethylene tubes (1 or 2 mm in diameter), in which the filling materials were inserted, the immersion medium of the filling materials (distilled water or PBS) on the outcomes of solubility, and micro-CT analysis. (4) The fourth objective was to assess the surface element distribution on the material and the crystalline phases of Calen-ZO and Bio-CP with dispersive X-ray spectroscopy (SEM-EDX) and X-ray diffraction (XRD), when immersed in PBS. The null hypothesis was that there would be no difference among materials for all parameters.

## 2. Materials and Methods

Sample size calculation was determined using *G* ^*∗*^Power 3.1 software for Windows (Franz Faul, Christian-Albrechts-Universität zu Kiel, Kiel, Germany). The calculation for each of the outcomes using an effect size based on pilot tests, at least 0.8 test power (*β*), and *α* = 0.05, using “*F* test family” (one-way ANOVA), showed that *n* = 10 would be necessary for each material, polyethylene tube, and immersion medium groups. However, as losses could occur, *n* = 12 was used.

Root filling materials, manufacturers, and proportions for manipulation are shown in Table 1 [3].

### 2.1. Solubility Test

The primary objective of this study was to develop a new methodology based on the principles outlined in ISO 6876 standard. Transparent cylindrical polyethylene tubes (Embramed, Cremer, São Paulo, SP, Brazil) measuring 1 or 2 mm diameter and 13 mm long, were closed at one end, so that the final length was 10 mm (*n* = 12 for each material, polyethylene tube group, and immersion medium). ZOE was inserted and condensed inside the polyethylene tubes using Paiva condensers (Golgran, São Caetano do Sul, SP, Brazil). Gentle compaction movements were applied until the tube was completely filled without any visible voids. Calen-ZO was placed inside a 3-mm syringe (Becton Dickinson, Curitiba, PR) and applied with a 21G 0.80 × 30-mm needle (Becton Dickinson), to allow slow insertion into the polyethylene tubes [12]. Bio-CP, available in a ready-to-use syringe, was slowly inserted into the polyethylene tubes using the manufacturer-provided tips. Ultrasonic activation was made with ultrasonic tip E1 – Irrisonic (Helse Ultrasonics, Santa Rosa de Viterbo, SP, Brazil), by using the ultrasonic Ultrawave XS device (Ultradent, Indaiatuba, SP, Brazil) at 20% power to obtain homogeneous, void-free tube filling. Radiographs of all the specimens were taken to determine the quality of the filling. The specimens were weighed, and the value was recorded as the initial mass. Immediately after, they were immersed in a flask containing 10 mL of PBS or distilled water and stored in a lab oven at 37°C for 30 days [9]. Then, they were removed from the flasks and placed in an oven at 37°C, supplemented with silica, until the specimens' mass stabilized. Afterward, the specimens were kept at room temperature before final weighing. The value was recorded as final mass value. The mass loss was determined by subtracting the initial from the final weight values, expressed as a percentage.

### 2.2. Volumetric Change, Voids, and Filling Capacity

Another set of polyethylene tubes was used for this test (*n* = 12), as described hereinabove. After the tubes were filled with the materials, and the quality of the filling was established by radiographs, the tubes were immediately scanned by micro-CT (SkyScan 1176; Bruker-micro-CT, Kontich, Belgium). The micro-CT parameters were 18 *μ*m voxel size, 80 kV, 125 *μ*A, rotation step 0.5, frame 4, 1.0 mm aluminum filter, and 180° scanning evolution cycle. The scanning time for every two specimens was 23–25 min. The reconstruction of the images was performed using NRecon software (V1.6.10.4; Bruker Micro-CT). The correction parameters for smoothing, beam hardening, and ring artifacts were defined for each material as follows: for Bio-CP, 0 for beam hardening, 4 for ring reduction, and 0 for smoothing; for Calen-ZO, 20 for beam hardening, 5 for ring reduction, and 0 for smoothing; and for ZOE, 50 for beam hardening, 5 for ring reduction, and 0 for smoothing. After micro-CT scanning, the specimens were immersed in a flask containing 10 mL of PBS or distilled water and stored in a lab oven at 37°C for 30 days [9]. The specimens were removed from the flasks and immediately rescanned, using the same acquisition parameters used in the initial scan [8]. The initial reconstructed images were superimposed on the reconstructed images after the solubility challenge, by using Data Viewer software (V1.5.2.4; Bruker Micro-CT). Micro-CT analysis included the total volume (mm^3^) of the root filling material, the total volume of the material in the established region of interest (ROI) (% filling capacity), and the voids (mm^3^), which were calculated by CTAn software (V1.15.4.0; Bruker Micro-CT). Volumetric alteration and voids were expressed as the root filling material volume either gained or lost, as a percentage [8, 9, 13].

### 2.3. Material Surface Element Distribution: SEM-EDS Analysis

This test was performed to detect hydroxyapatite precipitate. Another set of polyethylene tubes (2 mm in diameter) (Embramed) was used for this test (*n* = 3 for each material (Bio-CP and Calen-ZO), polyethylene tube group, and immersion medium), as described hereinabove. The materials were placed in the tubes and immersed in PBS for 14 or 30 days inside plastic flasks containing 10 mL of the solution and stored in a lab oven at 37°C. Immediately afterward, the specimens were desiccated for 14 days in a lab oven at 37°C. Afterward, the tubes were mounted in SEM stubs, with the open end pointing upward. Distribution of the surface element on the specimens was assessed by scanning electron microscopy (SEM, JSM 6610LV, JEOL, Musashino, Akishima, Tokyo, Japan) at 25 kV and ×100 magnification, and 10 mm working distance. The energy-dispersive spectroscopy (EDS, Oxford Instruments X-Max, OIC Precision Labs, Winnipeg, Manitoba, Canada) was performed without a carbon coating process.

### 2.4. Crystalline Phase Identification: XRD Analysis

This test was performed to detect hydroxyapatite components. The same specimens used for SEM-EDS analysis were used for XRD. The surface of each specimen was carefully scraped and crushed to a fine powder, and the powder was then spread evenly in the holder. An automated X-ray diffractometer (D2 PHASER; Bruker-AXS, Karlsruhe, Germany) was applied using the scan parameters set at steps of 0.02° every 0.2 s and analyzed between 10° and 80° 2*θ*; each analysis took about 12 min. Origin 2022 for Windows OS (OriginLab Corporation, Northampton, MA, USA) was used to perform the qualitative analysis of the diffractogram. The peaks of each sample were matched with those of the International Center for Diffraction Data (ICDD) database (International Center for Diffraction Data, Newton Square, PA, USA).

### 2.5. Statistical Analysis

The data were evaluated using GraphPad Prism 9.0 statistical software for Windows OS (GraphPad Software, San Diego, CA, USA). The Shapiro–Wilk test showed that the voids and solubility data did not fully comply with a normal distribution; therefore, Kruskal–Wallis followed by the Dunn's test was used to detect the differences among the groups. Two-way ANOVA and the Tukey post hoc test were used to determine the volumetric change and filling capacity (*α* = 0.05).

## 3. Results

**Figure 1:**
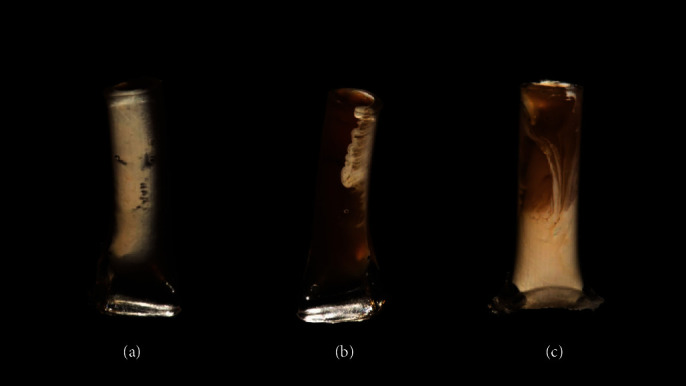
Front (a, c) and back (b) views of polyethylene tubes filled with Bio-CP left to rest for 45 min after filling. The images show the separation into two phases of Bio-CP.

The Bio-CP results were compared statistically with the other materials only in the solubility and filling capacity analyses (Tables 2 and 3). Bio-CP results are shown in Tables 3 and 4 but were not considered in the comparisons for volumetric change or presence of voids, because the Bio-CP separated into two phases during the micro-CT scanning time (23–25 min), as shown in Figure 1.

### 3.1. Solubility

In distilled water, ZOE was more soluble than Bio-CP and Calen-ZO in both 1 and 2-mm tube diameters (*P* < 0.05). Bio-CP and Calen-ZO were not different in 1-mm tube diameter (*P* > 0.05). Calen-ZO was more soluble than Bio-CP (*P*=.0157) in 2-mm-diameter tubes. In PBS, in both 1 and 2-mm tube diameters, ZOE was more soluble than Bio-CP and Calen-ZO (*P* < 0.05), which were not different between them (*P* < 0.05).

In both tube diameters (1 and 2 mm), Bio-CP and Calen-ZO were more soluble in water than in PBS (*P* < 0.05), while ZOE showed no difference regardless of immersion medium (*P* > 0.05) but had higher solubility rates than the other materials (*P* < 0.0001). In 2-mm tube diameter, all the materials showed greater solubility regardless of immersion medium, except for Bio-CP in distilled water, which showed no difference (*P*=0.2768) between both tube diameters (1 and 2 mm) (Table 2).

### 3.2. Filling Capacity

**Figure 2:**
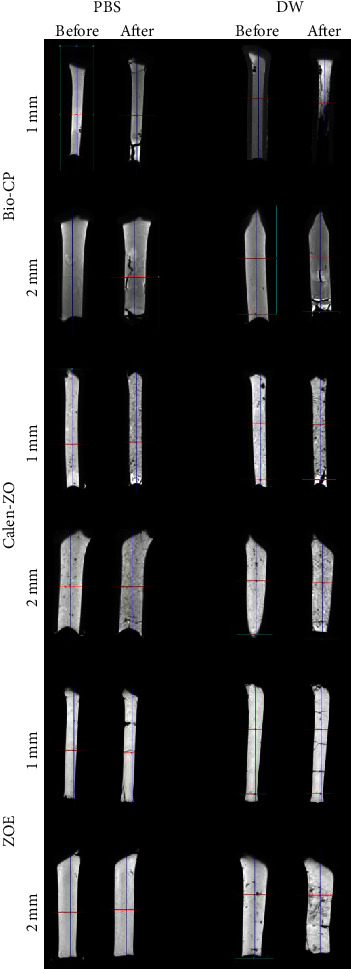
Representative micro-CT images showing filling capacity, volumetric change, and voids of Bio-CP, Calen-ZO, and ZOE in 1- or 2-mm polyethylene tubes after immersion in distilled water or PBS for 30 days. The “before” micro-CT images represent filling capacity. The “before” and “after” micro-CT images represent volumetric change and voids.

There was no statistical difference among the materials regardless of tube diameter (*P* > 0.05) (Table 3 and Figure 2).

### 3.3. Volumetric Change

All the materials showed volumetric loss. In water, Calen-ZO had more volumetric loss than ZOE (*P* < 0.05) regardless of tube diameter. In PBS, there was no difference between Calen-ZO and ZOE (*P* > 0.05) regardless of tube diameter.

In 2-mm tube diameter, Calen-ZO showed greater volumetric loss in distilled water than PBS (*P* < 0.05). Additionally, in distilled water, Calen-ZO showed greater volumetric loss in 2-mm tube diameter than in 1-mm tube diameter (*P* < 0.05) (Table 4 and Figure 2).

### 3.4. Voids

In water, there was no difference between Calen-ZO and ZOE (*P* > 0.05), regardless of tube diameter, except in 1-mm tube diameter, wherein Calen-ZO had more voids than ZOE (*P* < 0.05). In PBS, Calen-ZO and ZOE showed no differences between them (*P* > 0.05), regardless of tube diameter.

In 2-mm tube diameter, Calen-ZO showed greater presence of voids in distilled water than in PBS (*P* < 0.05). ZOE showed no differences in distilled water and PBS, regardless of the tube diameter (Table 5 and Figure 2).

### 3.5. Material Surface Element Distribution

**Figure 3:**
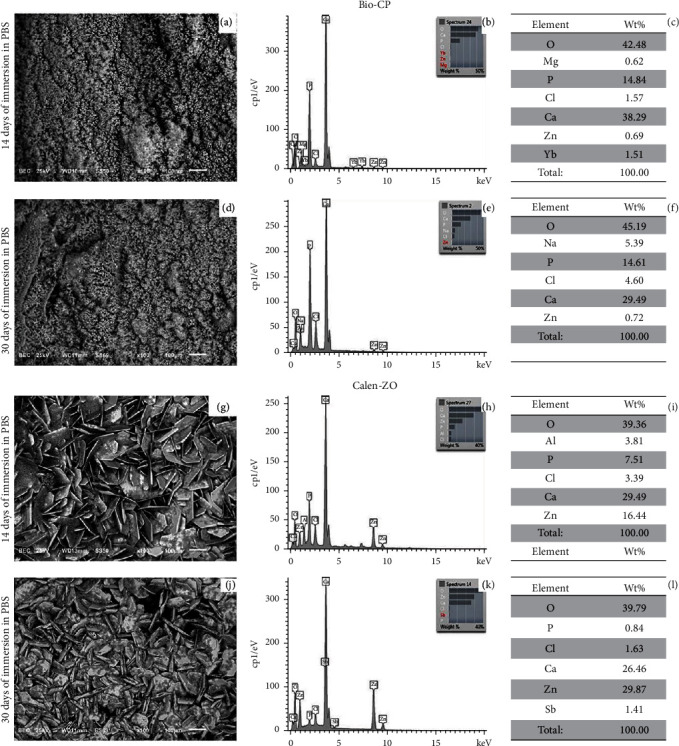
SEM examination of surface structure of Bio-CP (a–d) and Calen-ZO (g–j); EDX analysis of Bio-CP (b–e) and Calen-ZO (h–k); and element identification of Bio-CP (c–f) and Calen-ZO (i–l) after immersion in PBS for 14 and 30 days. Barr = 100 *µ*m.

The EDX detected oxygen (O), magnesium (Mg), phosphorous (P), chlorine (Cl), calcium (Ca), zinc (Zn), and ytterbium (Yb) on the Bio-CP specimens immersed in PBS for 14 days. Apart from these elements detected at 14 days, sodium (Na) was also detected when the specimens were immersed in PBS for 30 days (Figure 3).

The EDX detected oxygen (O), aluminum (Al), phosphorous (P), chlorine (Cl), calcium (Ca), and zinc (Zn) on the Calen-ZO specimens immersed in PBS for 14 days. The surface of Calen-ZO specimens showed crystalline phase formations after immersion in PBS. The absence of Al and the presence of antimony (Sb) was detected when the specimens were immersed in PBS for 30 days (Figure 3). The elements identified in the crystalline formations were comparable to those identified in the total surface analysis.

The morphology analysis of the Calen-ZO surface showed the characteristic crystal-like plate formation. In contrast, the surface of Bio-CP showed urchin-like crystallite structure.

### 3.6. Crystalline Phase Identification: XRD Analysis

**Figure 4:**
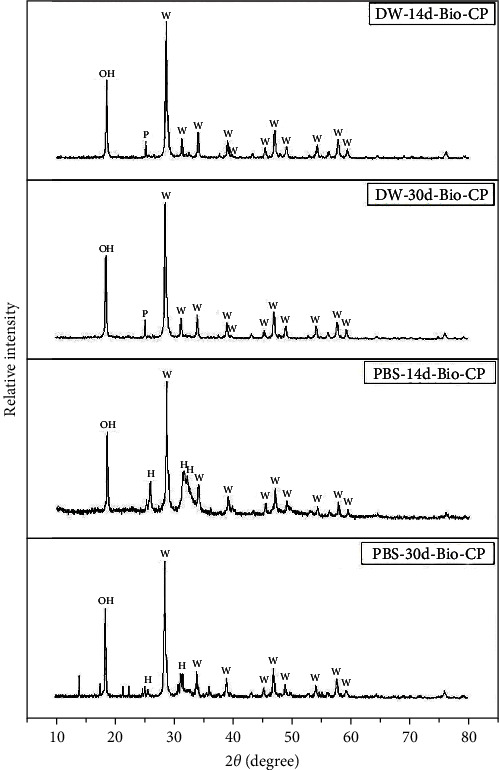
X-ray diffractogram (XRD). For DW-14d-Bio-CP and DW-30d-Bio-CP, the formation of peaks can be identified, referring to the presence of calcium tungstate (W), doyleite (OH) and calcium phosphate (P) crystal phases. For PBS-14d-Bio-CP and PBS-30d-Bio-CP, carbonated hydroxyapatite (H), calcium tungstate (W) and doyleite (OH) crystal phases can be identified.

**Figure 5:**
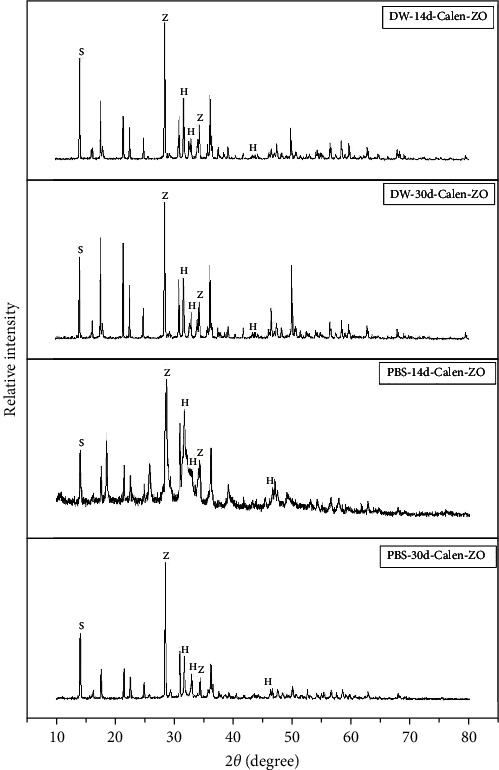
X-ray diffractogram (XRD). For DW-14d-Calen-ZO and DW-30d-Calen-ZO, the formation of peaks can be identified, referring to the presence of carbonated hydroxyapatite (H), zinc oxide (Z), and calcium silicate (S) crystal phases. For PBS-14d-Calen-ZO and PBS-30d-Calen-ZO, carbonated hydroxyapatite (H), zinc oxide (Z) and calcium silicate (S) crystal phases can be identified.

After 14 days of immersion of the Bio-CP specimens in PBS, XRD analysis detected carbonate hydroxyapatite (C0.22 Ca5 O13.514 P2.823; 96-900-3552), calcium tungstate (CaOW4; 96-721-9230), and doyleite (Al(OH)3; 1980-041). After 30 days of immersion, the same crystal phases were identified in Bio-CP (Figure 4). Calen-ZO showed carbonate hydroxyapatite (C0.22 Ca5 O13.514 P2.823; 96-900-3552), zinc oxide (ZnO; 96-230-0113), and calcium silicate (Ca2SiO4; 1344-95-2) after 14 and 30 days of immersion in PBS (Figure 5).

## 4. Discussion

The current study evaluated solubility, volumetric change, and presence of voids in the Bio-CP, Calen-ZO, and ZOE materials inserted in polyethylene tubes 1 or 2 mm in diameter and immersed in PBS or distilled water. The distribution of the chemical elements and the crystalline phases on the surface of Calen-ZO and Bio-CP was also evaluated after immersion in PBS. The null hypothesis was rejected since differences were observed among materials.

The present study aimed to develop a new methodology based on the principles outlined in ISO 6876 standard. To achieve this, we conducted experiments over a 30-day period using two immersion mediums—PBS and water—to comprehensively assess materials behavior. Traditionally, the solubility of root canal sealers is evaluated according to ISO 6876 standard, which stipulate that it should not be over 3% after 24 hr of immersion of the sealer in water. In the present study, a period of 30 days and two immersion mediums, PBS and water, were applied to gain a better understanding of the behavior of the materials and improve the clinical relevance of the results [8, 9]. Polyethylene tubes closed at one end were used to achieve a better simulation of a clinical situation [12], insofar as Bio-CP and Calen-ZO do not set [3]. Some researchers have used prototyped 3D printed teeth to evaluate instrumentation protocols for primary [14, 15] and permanent teeth [16]. However, these prototyped teeth have a radiopacity similar to dentin. This could create a ROI delimitation problem when using micro-CT software, because Calen-ZO and Bio-CP have a radiopacity close to 3 mm Al [3], hence similar to the prototyped tooth. In addition, depending on the 3D printer, defects caused by printing resolution inaccuracies could be identified at the internal root canal surface [15] and thus impair the micro-CT analysis. Another alternative would be to use natural teeth, but this option was discarded, because it was difficult to obtain natural primary teeth with little root resorption. Moreover, no interaction between the filling materials and dentin was evaluated in this study, thereby giving preference to polyethylene tubes.

Filling materials for primary teeth root canals should accompany the physiological resorption of the primary tooth roots [17]. However, there is no definite value or study that correlates physiological root resorption speed with the solubility of the root canal filling materials. Bioceramic materials used for endodontic permanent tooth treatment are highly soluble [10], reported by ISO 6876 as being more than 3%. However, this 3% solubility rate cannot be used as a parameter for analyzing the root canal filling materials for primary teeth.

All the study materials evaluated had more than 7% solubility, regardless of the immersion medium or the tube diameter. There was no difference between Bio-CP and Calen-ZO, and both were less soluble than ZOE in both immersion mediums. A recent study showed that Calen-ZO, inserted in 1-mm-diameter polyethylene tubes, had higher solubility than ZOE immersed in water for 35 days. Calen-ZO and ZOE had a solubility rate of 22.05% and 12.65%, respectively [18], compared with 10.54% (Calen-ZO) and 16.57% (ZOE) in 1-mm-diameter tubes in the present study. There are three possible explanations for the different results of Pintor et al. [18]. (1) Although not mentioned, they probably waited for ZOE to set before immersing it in water, unlike the present study, in which the specimens were immersed immediately after handling. Studies show that after ZOE sets, it has a solubility rate of approximately 4.5% after 30 days of immersion in distilled water [19]. (2) According to the materials studied, Pintor et al. [18] used Calen in its commercial formulation, without adding zinc oxide; that is, it was a more flowable alternative. (3) The authors removed the specimens, placed them in a desiccator for 24 hr, weighed them, and then reimmersed them in water up to the next experimental time point (7, 21, 28, and 35 days). Another study showed that Bio-CP had greater solubility than ZOE [6]. The divergent results could be attributed to differences in methodologies since those authors used material disks rather than inserting the material into tubes. Additionally, information regarding the ZOE setting time or the immediate immersion of disks in water was not provided.

Bio-CP and Calen-ZO had significantly lower solubility rates in PBS compared with distilled water. The lower solubility of Bio-CP in PBS than in water can be explained by the formation of apatite-like structures on the surface of calcium silicate-based endodontic materials when in contact with phosphate buffered solutions [11, 20], which could potentially slow down their solubilization process. In relation to Calen-ZO, a calcium hydroxide-based material, Ca(OH)_2_ undergoes a chemical reaction with the phosphate ions present in the tissue fluids, resulting in hydroxyapatite formation on the material surface [21, 22, 23, 24]. Hydroxyapatite formation in Bio-CP and Calen-ZO immersed in PBS was confirmed in both materials by XRD analysis. SEM results evidenced that Bio-CP promoted an urchin-like crystallite structure, which has also been observed in a previous study that used PBS [6]. ZOE was not included in EDX and XDR analyses because a previous study demonstrated that after immersion in physiological solution, the only elements detected in IRM (a ZOE-based cement) are zinc and carbon, which are not related to hydroxyapatite formation [25]. On the other hand, it is important to mention that hydroxyapatite formation in a laboratory cannot be extrapolated directly to the clinical scenario, since the formation of crystals in in vitro models may represent an overestimation of the in vivo conditions [20, 26]. Regarding the tube diameter, all materials showed more solubility in 2-mm tubes both in PBS and in water. This is probably related to the difference in surface contact with the immersion mediums (1 vs. 2 mm diameter).

Regarding solubility test, it may be questioned whether washing could be considered a risk of bias in materials that do not set (Bio-C and Calen-ZO) as they could be washed out and displaced from the tube. Although this washout was monitored macroscopically not to occur, it may have been possible for it to occur microscopically. For this reason, the solubility test was complemented with the analysis of volumetric changes by micro-CT.

The divergent results between solubility and volumetric changes tests could be due to differences in testing procedures. In the case of micro-CT, polyethylene tubes were filled with materials and then submitted to scanning, which took approximately 25 min. During this time, ZOE began initial setting process, resulting in minimal volumetric changes before immersion in distilled water or PBS during 30 days. Conversely, in an attempt to simulate a clinical situation, during solubility test, the polyethylene tubes were filled with materials and immediately immersed in either distilled water or PBS. This procedure prevented ZOE to set. Consequently, ZOE remained unstable while immersed, which led to higher solubility.

Micro-CT scanning is a high-resolution, nondestructive method that allows the quantification of the volumetric change and voids and a tridimensional evaluation of the quality of the filling [27]. In the analyses of volumetric change and voids, Bio-CP was not considered in the comparison between materials, because it separated into two phases after the scanning time (23–25 min), and a small portion of the material had leaked out of the tube in some specimens. It could be questioned why the high fluidity of Bio-CP allowed leakage out of the tube during microtomography but none during the solubility test. During the solubility test, the immersion media were in intimate contact with the Bio-CP. Although this material is fluid, the different density with water or PBS, or even differences in pressure prevented the material from leaking from the tube. Contrarily, during the micro-CT scans, there was no immersion medium, which allowed the fluidity of the Bio-CP to promote leakage.

ZOE had a lower volumetric loss than Calen-ZO in distilled water, and there was no difference in the volumetric change of either material when immersed in PBS. Considering the solubility test results, ZOE was expected to have a greater volumetric loss than Calen-ZO in water. However, in the tests with micro-CT, the materials were not immersed in water or PBS immediately after manipulation, since the scanning time for every two specimens was 23–25 min. During this time, partial setting of ZOE may have occurred (ZOE has a setting time of 110 min) [3], leading to lower mass loss than Calen-ZO in water, since Calen-ZO does not set [28]. The lack of a difference between Calen-ZO and ZOE in PBS and the lower volumetric change of Calen-ZO immersed in PBS than water are probably due to the formation of hydroxyapatite, which delays the solubility of Calen-ZO [21, 22, 23].

Endodontic materials should not have a high percentage of voids, which may increase post-treatment apical periodontitis [27]. The occurrence of voids in the root filling may be influenced by several factors, such as the consistency and volume of the material, the operator's expertise, and the filling technique used [29]. In the present study, polyethylene tubes closed at one end were filled by a single operator using the obturation technique recommended for each material. The only difference in the void formation between Calen-ZO and ZOE occurred with the 1-mm tube diameter immersed in distilled water, where Calen-ZO showed higher presence of voids than ZOE.

It is important to mention that even though Bio-CP is available in a ready-to-use syringe, the ultrasonic tip had to be used to ensure homogeneous filling of the tubes without the presence of voids (detected radiographically), which formed when the material was inserted in the polyethylene tubes through the syringe and application tip. Ultrasonic activation can be used to agitate the intracanal sealers and intracanal medication pastes. This enhances the intratubular dentin penetration of the sealer, thus promoting antimicrobial activity against *Enterococcus faecalis* [30] and minimizing the presence of gaps in vitro [31].

The filling capacity of all materials was higher than 98%, regardless of the obturation technique applied. In the present study, the filling capacity was assessed by micro-CT; however, other methodologies such as digital microscopy, confocal laser scanning microscopy analysis (CLSM), or even SEM could also be used, as previously described [32]. It is noteworthy that the phase separation of Bio-CP caused by the chemical instability of the material did not influence the filling capacity percentage. Nonetheless, the composition of Bio-CP must be revised to achieve better chemical stability and filling capacity, without having to use ultrasonics. A recent study compared filling effectiveness, root filling voids, and obturation techniques in resin-prototyped primary incisors, by using micro-CT. When the authors used a lentulo to obturate the incisor with Calen-ZO, they found a filling capacity of 94.97%, compared with 99.77% when they used a syringe [33]. These results are comparable to those of the present study, which used a syringe, namely, 98.87% for 1-mm-diameter tubes, and 98.42% for 2-mm tube diameter. In the case of ZOE, there was a 96.35% filling capacity using a lentulo, versus 93.45% using a syringe [33], whereas the filling capacity in our study was higher than 98% for 1- and 2-mm tube diameters.

The present study sought an adequate in vitro methodology based on ISO 6876 standard to evaluate the solubility (% of mass loss) and the volumetric and morphological alterations of root canal fillings for primary teeth. It found that insertion of the fillings into 1- or 2-mm-diameter tubes, followed by 30-day immersion in water or PBS, allowed evaluation of the solubility, volumetric change, and voids of root canal filling materials for primary teeth. However, when one of the study materials is based on calcium silicate or calcium hydroxide, the immersion medium of choice should be PBS, owing to the formation of hydroxyapatite-like structures that can influence the solubility [21, 23]. Regarding the tube diameter, in most cases, the materials inserted in 1-mm tube diameter showed lower solubility than those inserted in 2-mm tube diameter. Further studies should be conducted to correlate the solubility of the materials, their resorption speed, and the rhizolysis speed in primary teeth, in order to define the percentage of solubility that root canal filling materials should have. Theoretically, 1-mm tube diameter simulates primary molar teeth better, and 2-mm tube diameter simulates primary anterior teeth better. The micro-CT analysis poses limitations in comparing materials that do not set with materials that do, as in the case of Calen-ZO and ZOE, respectively. That is because, even though materials that set may be placed inside the tubes and analyzed by micro-CT immediately after handling, they would eventually set either fully or at least partially, depending on their setting time. Thus, they should be placed in the immersion medium after setting to avoid creating bias regarding the comparison the materials.

Finally, it is important to consider that the findings of in vitro studies cannot be extrapolated to a clinical situation due to inherent limitations such as not evaluating the biological interaction, which could be evaluated in in vivo studies [34].

## 5. Conclusion

Overall, it can be concluded that there was no difference between the solubility of Bio-CP versus Calen-ZO, and that both had lower solubility than ZOE, regardless of the immersion medium (distilled water or PBS) and tube diameter (1 or 2 mm). Bio-CP and Calen-ZO produced hydroxyapatite after 14 days of immersion in PBS. Micro-CT analysis of volumetric change and voids is not recommended for Bio-CP, because it separates into two phases, thus creating a bias regarding the comparison of the materials. There was no difference in volumetric loss or void percentage between Calen-ZO and ZOE when immersed in PBS. From a clinical perspective, Bio-CP has the potential to become a suitable material for root canal filling in primary teeth. Nonetheless, its composition must first be revised to achieve better chemical stability prior to a widespread recommendation.

## Figures and Tables

**Table 1:** Root canal filling material, manufacturer, and proportion of use.

Material	Manufacturer	Proportion for manipulation
Bio-C Pulpecto ^*∗*^	Angelus Indústria de Produtos Odontológicos S/A, Londrina, PR, Brazil	Ready for use

Calen + zinc oxide (Calen-ZO)	(Calen) S.S. White Artigos Dentários Ltda., Rio de Janeiro, RJ, Brazil (Zinc oxide) Biodinâmica Química e Farmacêutica LTDA, Ibiporã, PR, Brazil	0.24 g Calen + 0.12 g ZO

Zinc oxide and eugenol (ZOE)	Biodinâmica Química e Farmacêutica	0.54 g ZO + 100 *μ*L of eugenol

^*∗*^Product in development, batch no. 190118.

**Table 2:** Solubility comparison (% mass loss) of the materials inserted in polyethylene tubes (1 or 2 mm diameter) and immersed in distilled water (DW) or PBS.

Materials	1 mm diameter		2 mm diameter	
	DW	PBS	DW	PBS
	M (Q1–Q3)	M (Q1–Q3)	M (Q1–Q3)	M (Q1–Q3)
Bio-CP	9.206 (−8.459–10.14)^A ^*∗*^^	7.524 (7.427–7.931)^A#^	9.70 (9.521–9.73)^A ^*∗*^^	8.480 (8.126–8.965)^A^
Calen-ZO	10.54 (−8.0–11.46)^A ^*∗*^#^	7.554 (6.877–8.224)^A#^	13.03 (12.59–14.08)^B ^*∗*^^	9.592 (9.129–9.664)^A^
ZOE	16.57 (15.59–17.50)^B#^	17.82 (14.20–18.06)^B#^	22.35 (22.16–23.06)^C^	20.45 (20.36–23.32)^B^

The values are expressed as M (Q1–Q3); M, median; Q1, first quartile; Q3, third quartile. Different uppercase letters (A, B, and C) in the columns indicate statistically significant difference (*P* < 0.05) among materials in each immersion medium;  ^*∗*^ indicates statistically significant difference (*P* < 0.05) between immersion mediums for each material of the same polyethylene tube diameter (1 or 2 mm); ^#^ indicates statistically significant difference (*P* < 0.05) between polyethylene tube diameter (1 or 2 mm) in the same immersion medium for each material (statistical analysis using the Kruskal–Wallis and Dunn's tests).

**Table 3:** Filling capacity (in %) of the materials inserted in polyethylene tubes (1 or 2 mm diameter).

Materials	1 mm diameter	2 mm diameter
	M (Q1–Q3)	M (Q1–Q3)
Bio-CP	99.30 (98.47–99.63)	99.13 (98.34–99.42)
Calen-ZO	98.87 (97.84–99.15)	98.42 (97.20–99.17)
ZOE	98.44 (97.87–98.98)	98.07 (97.01–98.76)

The values are expressed as M (Q1–Q3); M, median; Q1, first quartile; Q3, third quartile. The absence of letters indicates no statistical difference between materials in each polyethylene tube diameter (columns) and between 1- and 2-mm tube diameter for each material (rows) (*P* > 0.05) (statistical analysis using two-way ANOVA and Tukey's post-test, *α* = 0.05).

**Table 4:** Volumetric change (%) of the materials inserted in polyethylene tubes (1 or 2 mm diameter) and immersed in distilled water (DW) or PBS.

Materials	1 mm diameter		2 mm diameter	
	DW	PBS	DW	PBS
	Mean (SD)	Mean (SD)	Mean (SD)	Mean (SD)
Bio-CP	−35.89 (30.43)	−5.573 (2.529)	−25.36 (14.27)	−5.658 (2.406)
Calen-ZO	−4.711 (2.954)^A#^	−2.296 (1.032)^A^	−15.14 (8.886)^A^ ^*∗*^	−1.930 (0.853)^A^
ZOE	−0.921 (0.805)^B^	−1.810 (1.813)^A^	−5.048 (6.952)^B^	−1.597 (1.274)^A^

Bio-CP results were not considered in the statistical comparisons. Values were expressed in mean and standard deviation (SD). Negative sign indicates volumetric loss. Different uppercase letters (A and B) in the columns indicate statistically significant difference (*P* < 0.05) between materials in each immersion medium; ^ ^*∗*^^ indicates statistically significant difference (*P* < 0.05) between immersion mediums for each material of the same polyethylene tube diameter (1 or 2 mm); ^#^ indicates statistically significant difference (*P* < 0.05) between polyethylene tube diameter (1 or 2 mm) in the same immersion medium for each material (statistical analysis using two-way ANOVA and Tukey's post-test, *α* = 0.05).

**Table 5:** Void (%) comparison of the materials inserted in polyethylene tubes (1 or 2 mm diameter) and immersed in distilled water (DW) or PBS.

Material	1 mm diameter		2 mm diameter	
	DW	PBS	DW	PBS
	M (Q1–Q3)	M (Q1–Q3)	M (Q1–Q3)	M (Q1–Q3)
Bio-CP	2115 (562.2–12,199)	629.2 (282.7–6,294)	579.8 (325.4–6033)	179.7 (−54.68–583.4)
Calen-ZO	398.9 (302.3–475.8)^A^	275.7 (194.7–363.4)^A^	525.6 (229.5–829.6)^A ^*∗*^^	116.8 (97.26–146.8)^A^
ZOE	41.37 (29.72–135.8)^B^	219.3 (127.4–311.2)^A^	203.7 (67.85–507.9)^A^	93.94 (77.94–195.4)^A^

Bio-CP results were not considered in the statistical comparisons. The values are expressed as M (Q1–Q3); M, median; Q1, first quartile; Q3, third quartile. Different uppercase letters (A and B) in the columns indicate statistically significant difference (*P* < 0.05) between the materials in each immersion medium;  ^ ^*∗*^^ indicates statistically significant difference (*P* < 0.05) between immersion mediums for each material of the same polyethylene tube diameter (1 or 2 mm) (statistical analysis using Kruskal–Wallis and Dunn's test).

## Data Availability

The data used to support the findings of this study are available from the corresponding author upon request.
